# Senescent tumor cells: an overlooked adversary in the battle against cancer

**DOI:** 10.1038/s12276-021-00717-5

**Published:** 2021-12-16

**Authors:** Soon Sang Park, Yong Won Choi, Jang-Hee Kim, Hong Seok Kim, Tae Jun Park

**Affiliations:** 1 Department of Biochemistry and Molecular Biology, Ajou University School of Medicine, Suwon, 16499 Korea; 2 Department of Biomedical Sciences, Ajou University Graduate School of Medicine, Suwon, 16499 Korea; 3 Inflamm-Aging Translational Research Center, Ajou University School of Medicine, Suwon, 16499 Korea; 4 Department of Hematology–Oncology, Ajou University School of Medicine, Suwon, 16499 Korea; 5 Department of Pathology, Ajou University School of Medicine, Suwon, 16499 Korea; 6 Department of Molecular Medicine, Inha University College of Medicine, Incheon, 22212 Korea

**Keywords:** Cancer microenvironment, Cancer immunotherapy

## Abstract

Senescent cells in cancer tissue, including senescent fibroblasts and macrophages, have been reported to increase the malignant potency of cancer cells by secreting senescence-associated secretory phenotype (SASP). Otherwise, Senescence of tumor cells has been believed to inhibit tumor growth by halting the massive proliferation and increasing the chances of immune clearance. In particular, senescent tumor cells (STCs) have been thought that they rarely exist in carcinomas because oncogene-induced senescence needs to be overcome for protumorigenic cells to become malignant. However, recent studies have revealed that a considerable number of STCs are present in cancer tissue, even in metastatic sites. In fact, STCs are widely involved in cancer progression by leading to collective invasion and building a cytokine barrier to protect nonsenescent tumor cells from immune attack. Furthermore, therapy-induced STCs can induce tumor progression and recurrence by increasing stemness. However, obscure causative factors and their heterogeneity in various cancers make it difficult to establish the physiological role of STCs. Here, we summarize and review the current knowledge of the pathophysiology and role of STCs. We also outline the current status of therapeutic strategies for directly removing STCs or modulating the SASPs to maximize the positive functions of STCs while suppressing the negative functions.

## Cancer: Non-dividing tumor cells offer a promising therapeutic target

Cancer cells that stop dividing but do not die help fuel tumor growth, and therapies that selectively eliminate or suppress these cells could reduce the risk of tumor recurrence and progression. A team from South Korea led by Hong Seok Kim from Inha University College of Medicine in Incheon and Tae Jun Park from Ajou University School of Medicine in Suwon review the cancer-promoting properties of tumor cells in irreversible cell cycle arrest, a state known as cellular senescence. They discuss how to identify these cells in cancer tissues and summarize the various drivers of senescence in the tumor microenvironment. Once established, the cells release local factors that both promote the invasiveness of nearby nonsenescent cancer cells and shield tumors from immune attack. Drugs that remove or modulate senescent tumor cells are now under investigation.

## Introduction

Cellular senescence is an irreversible cell cycle arrest caused by various internal or external stimuli^1–3^. The first reported type of cellular senescence was “replicative senescence” in the primary cell culture system^4,5^. The excess proliferating capacity of the cells and the shortening of their telomeres are significantly involved in replicative senescence^6,7^. In addition, triggering factors, including oncogene activation, DNA damage, or reactive oxygen species (ROS), can lead to early cellular senescence while maintaining replication capacity^8,9^. This type of cellular senescence is called “premature senescence”, and is observed in cancer cells^1,8,9^. Senescent cells feature overexpression of CDK inhibitors, such as p16^INK4A^, p21^WAF1^, and their master regulator p53^10–12^. Increased lysosomal senescence-associated β-galactosidase (SA-β-Gal) activity is another key marker of senescent cells^13,14^. Senescent cells generally undergo proinflammatory genomic reprogramming and release various types of cytokines and microvesicles, which is collectively called the senescence-associated secretory phenotype (SASP)^15,16^.

Cancer cells are the most proliferative cells in human-derived tissue. Paradoxically, senescent cells are frequently observed in cancer tissue^17–21^. Although various types of senescent cells are found in the tumor microenvironment, the most common type is senescent tumor cells (STCs)^22^. Cancer cells have a harsh environment, which is hypoxic and poorly nourished due to their insatiable desire for nutrients and oxygen, and these features easily lead to cellular damage^23,24^. The high replication burden of cancer cells also raises the possibility of genomic instability, which might be the main driver of cellular senescence^25^. However, until now, the exact mechanisms behind why and how cancer cells turn into STCs have remained unknown.

The importance of STCs in cancer progression has long been overlooked because they have been considered a product of a defense mechanism against cancer rather than a progression of it^26,27^. Many recent studies, however, have provided new insight into the cancer-promoting properties of STCs, such as their significant ability to promote local invasion and epithelial−mesenchymal transition (EMT)^17,28^. As remnant STCs following chemotherapy or radiotherapy are associated with cancer cell dormancy, which leads to a high rate of cancer relapse, controlling STCs after cancer treatment is important^29^. In this review, we present an overview of our current understanding of STCs. First, we offer appropriate markers to analyze STCs in human cancer tissues in vivo. Second, we summarize various drivers and causes of cancer cell senescence in the tumor microenvironment. Third, we provide the latest evidence that STCs promote cancer progression in a variety of cancers. Finally, we analyze the usability of senolytic drugs in cancer therapy.

## STCs in cancer tissues

Identifying the presence of senescent cells in human cancer tissue in vivo has challenges; however, the range of senescence markers has widened, making it easy to detect senescent cells^8^. Typically, the analysis of SA-β-Gal staining or p16^INK4A^ immunostaining are the most conventional and reliable methods to identify senescent cells used both in vitro and in vivo^8,13,30^. The trimethylation of lysine 9 in histone 3 (H3K9 trimethylation) can also be used as a senescence marker^20,31^. However, it is risky to detect senescent cells using a single marker considering the sensitivity and specificity of these markers. The major limitation of SA-β-Gal staining is its false positivity^14,30^. SA-β-Gal-positive cells are reported in normal tissues, such as the sebaceous gland and hair follicle tissues^14^. Even thick mucus in the gastrointestinal tract shows positive SA-β-Gal staining^32^. In the case of p16^INK4A^ immunohistochemistry (IHC), false positives occur in human papillomavirus infection or when the *CDKN2A* locus is mutated^33,34^. The requirement of nonfixed fresh frozen tissue for SA-β-gal staining because it is an enzymatic reaction is a major disadvantage preventing its use in large-scale studies of cellular senescence in human tissue. Alternatively, lipofuscin, which accumulates upon senescence induction and consists of lipid-containing residues of lysosomal digestion, can be detected by staining with Sudan Black B in formalin-fixed paraffin-embedded (FFPE) tissue^35^. Negative markers that should be absent in senescent cells can also be used. These markers are closely related to cell proliferation, such as Ki-67 or bromodeoxyuridine incorporation^36^. Instead of immunostaining, several methods have also been developed to identify senescence in ex vivo live tissues and organisms. For instance, the fluorescent senoprobes 5-dodecanoylaminofluorescein di-ß-D-galactopyranoside (C12FDG) and SPiDER-βGal have successfully been used to detect high β-galactosidase-expressing cells in live tissue by flow cytometry^37,38^. Nonetheless, these chromogenic fluorescent molecules and tracers have short wavelengths, resulting in the dispersion of fluorescence emission and leading to limited tissue penetrance^39^. To overcome this shortcoming, two-photon and near-infrared probes, including AHGa and NIR-BG, have been used to detect chemotherapy-induced senescence in vivo^40^. Furthermore, a positron emission tomography tracer called [18 F]FPyGal, the use of which indicated a close correlation between β-galactosidase activity and tracer uptake in chemotherapy-treated tumors in vivo, has also recently been developed^41^. Nevertheless, since various types of cells are mixed in tumor tissue, careful observation of histological slides is essential to identify the exact cell type.

**Fig. 1:**
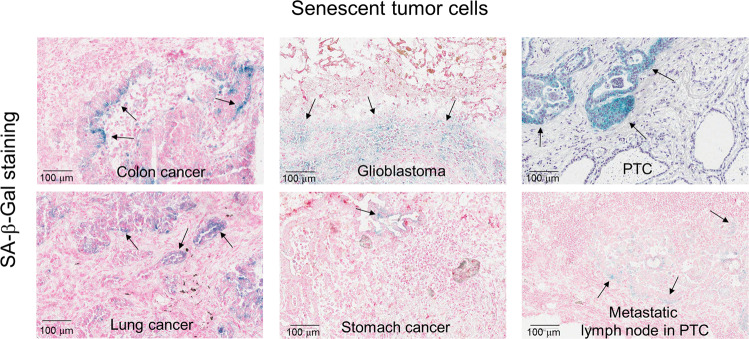
STCs in various cancer tissues. Multiple types of primary cancer tissue were processed to make fresh frozen sections for SA-β-Gal staining. Hematoxylin or nuclear fast red were used for counterstaining. Arrows indicate SA-β-Gal-positive STCs.

Although specific physiological roles are still lacking, three kinds of senescent cells have been observed in cancer tissues: tumor epithelial cells, macrophages, and fibroblasts^18^. Our previous studies have found that an equivalent number of SA-β-Gal-positive epithelial origin STCs exist in thyroid, stomach, and colorectal cancer and glioblastoma^17,18^ (Fig. 1). Costaining with epithelial cell markers showed that senescent cells are cancer epithelial cells^18^. These data suggest that some tumor cells turn into STCs during cancer progression. Unfortunately, STCs are morphologically indistinguishable from non-STCs in terms of tissue histology; enlarged cell size, and the formation of heterochromatin foci in the nucleus, which are commonly observed in in vitro senescent cells^8^, are not observed in vivo. While STCs are not distinct from non-STCs in appearance, their gene expression patterns differ. The expression of SASP markers, a major characteristic of senescent cells, is observed in STCs both in vivo and in vitro^1,16^. Significantly, the expression of SASP markers is thought to affect other types of surrounding cells in a paracrine manner^16,42^. Therefore, detecting STCs using representative SASP markers with conventional markers is effective for revealing the pathophysiological role of STCs. Another interesting feature of STCs is their local distribution in primary cancer. SA-β-Gal staining and p16^INK4A^ IHC analysis have revealed that STCs are often observed in the invasive region rather than in the center of cancer tissue^17,18^. STCs are thus thought to exist in the cancer invasive region to engage in cancer growth or inhibit immune cell infiltration. In addition, the representative senescent cell type in cancer tissue is macrophages. Round-shaped SA-β-Gal-positive cells, which are also CD68 positive, are often identified^18^. These cells commonly exist in intratumoral or peritumor regions^18^. CD68 and SA-β-Gal double-positive cells are also CD206 positive, which means they are M2-type macrophages^18,43^. Monocytes can be differentiated into M2 macrophages with several kinds of cytokine-induced signals^43,44^. The number of STCs and M2-type macrophages are proportional in colorectal cancer (CRC) tissue^18^. These data suggest that STCs are involved in M2-type macrophage differentiation. Finally, senescent cells observed in the tumor stroma and peritumoral region are vimentin-positive fibroblasts^18,44–47^. Our recent observations showed that senescent fibroblasts do not always exist in tumor tissues^18^. Furthermore, senescent fibroblasts did not show any correlation with STCs, but STCs showed a close correlation with senescent macrophages^18^. Senescent fibroblasts seem to be involved in the active proliferation of proneoplastic epithelial cells.

## Drivers of cellular senescence in cancer cells

Oncogene-induced senescence (OIS) is a potent tumor-suppressive mechanism that halts cell proliferation following potentially cancer-causing genetic alterations in normal cells^20,22^. The first observation of OIS was in an in vitro study in which an oncogenic form of Ras, H-Ras^G12V^, was ectopically expressed in the primary human lung fibroblast cell line IMR90^48^. Senescent growth arrest has also been stably established in in vivo animal models, with a notable example being a model featuring an ectopic expression of Ras in mammary epithelial cells^49^. Starting as studies of oncogenic Ras-induced senescence, OIS studies have gradually expanded to include the PI3K/AKT pathway, a signaling pathway that is related to approximately 40% of human cancers^50,51^. Mutant Ras-induced senescence is associated with the DNA damage response (DDR) caused by ROS, which leads to the recruitment and activation of the serine-threonine kinase ataxia-telangiectasia mutated^52–54^. On the other hand, cells undergoing AKT pathway-induced senescence do not display a DDR, DNA damage foci, or senescence-associated heterochromatin foci^55^. This type of senescence maintains low levels of p16^INK4A^ and phosphorylated p53^55^. Hyperactivation of the PI3K/AKT signaling pathway triggers senescence by promoting enhanced p53 protein synthesis *via* mTORC1^56^. Aberrant PI3K/AKT signaling also triggers the sequestration of MDM2 in the nucleolus in a PML/p19^ARF^-independent manner^56,57^. Despite the complexity of factors involved in OIS, it has been demonstrated that the tumor suppressors p53 and pRB are the two main regulators of OIS^20^. The loss of p53 or its regulator p19^ARF^ in mice provokes Ras-induced cancer cell invasion^58^; conversely, the reactivation of p53 suppresses tumor growth in association with the expression of common senescence markers^59^. Since OIS needs to be overcome for carcinoma development, OIS is mostly found in precancerous adenomatous lesions^60^. For instance, senescent cells are observed in H-Ras^G12V^ transgenic mouse squamous papilloma^61^. BRAF^V600E^-induced senescent melanocytes were also identified in mouse and human nevi^62^. Another possible example is human papillary thyroid carcinoma (PTC) containing the BRAF^V600E^ mutation, a crucial oncogene in the development of PTC^63,64^.

The most clinically common type of premature senescence is therapy-induced senescence (TIS). It is well established that STCs are easily triggered by chemotherapy^2,65^. The prosenescence activity of chemotherapeutics has been reported in various cancers, including breast, lung, prostate, and colorectal cancers^2,65^. In a previous study, approximately 40% of breast cancer patients who received neoadjuvant chemotherapy had SA-β-Gal- and p16^INK4A^-positive cancer cells^66^. In addition, non-small-cell lung cancer patients who received carboplatin and paclitaxel chemotherapy were found to often have STCs in their cancer tissue^67^. The major factor causing TIS by chemotherapy is extensive nuclear damage, such as DNA breaks and modification, induced by effects such as alkylation by genotoxic agents and ROS generation^65^. However, the induction of senescence by chemotherapy seems to be caused by a telomere-independent mechanism, as breast cancer cells exposed to doxorubicin did not show telomere shortening even though the accumulation of some cytogenetic changes within telomeres was found^68^. Ionizing radiation (IR) is another common therapeutic method that induces cellular senescence in various cancer cells^65^. A senescent phenotype induced by IR has been frequently observed in various cancer cell lines of in vitro culture systems and in in vivo mouse xenograft tumors^65^. Senescence caused by IR seems to be p53-dependent^69^; MDA-MB-231 breast cancer cells with attenuated p53 function fail to become senescent, ultimately leading to apoptosis. In glioma cells, PTEN deficiency results in senescence in damaged cancer cells, whereas sufficient PTEN expression leads the cells toward apoptosis^69^.

ROS is another important, well-known senescence inducer in vitro. Since OIS induces ROS generation through downstream signaling cascades, using H_2_O_2_ treatment to induce senescence in vitro is a well-established method^53^. In contrast, the role of ROS as senescence inducers in vivo has not been demonstrated. This is due to the short half-life of ROS and difficulties in measuring ROS in surgically removed cancer tissues^70^. The relative amount of ROS is indirectly quantified according to the expression of hypoxia-related or antioxidant proteins such as HIF-1α^71^. Although the exact mechanism is still disputed, it is suggested that STCs in the invasive region are closely related to ROS generation. The invasive region of cancer tissue is where cancer cells most massively proliferate. If angiogenesis does not follow, cancer cells are easily exposed to hypoxic insults^72^. Following abrupt revascularization with the angiogenetic activity of cancer cells may lead to the generation of a large amount of ROS^18,73^. In a study of CRC patients, p16^INK4A^ expression was inversely correlated with HIF-1α expression, which suggests that ROS generation following revascularization in the invasive front resulted in the generation of STCs^18^. In line with this, multiple studies have reported that HIF-1α-positive cancer cells are located in the invasive front of cancer tissue and are closely related to metastasis^74,75^. However, the evidence of a connection between tumor cell senescence and the hypoxic response is still weak; therefore, further studies on whether ROS can be the main inducer of tumor cell senescence should be performed.

## SASP expression in STCs

In addition to the presence of cell cycle arrest during the senescence process, senescent cells secrete a complex mixture of proteins collectively known as SASPs, a key non-cell-autonomous feature of senescence that distinguishes it from quiescence^1,3,22^. SASPs comprise a complex secretome of proinflammatory cytokines and chemokines, extracellular matrix proteins, growth factors, and matrix metalloproteinases, as well as exosome-like small extracellular vesicles^3,22,27^. NF-κB and C/EBP-β complexes, both of which are transcriptional machineries associated with inflammatory responses, are currently recognized as the main transcription factors globally regulating SASP expression^76^. Recent studies suggest that other pathways, including the GATA4, mTOR, and JAK2/STAT3 pathways, also play an important role in SASPs expression^77^. Although SASP expression differs between STCs, to our knowledge, the most vigorously expressed protumorigenic SASPs from STCs are (1) immune cell-modulating cytokines, (2) proangiogenic factors, and (3) extracellular matrix-modulating factors. In CRC cell lines, immune cell-modulating cytokines, including TNF-α, TGF-β, and various interleukin family molecules and chemokines, are significantly upregulated in H_2_O_2_-induced senescent cells^18^. Specifically, our previous study demonstrated that C-X-C motif chemokine 12 (CXCL12) is a key modulator of the antitumor function of cytotoxic T lymphocytes^18^. Another potent key immune-modulating SASP, IL-6, recruits myeloid-derived suppressor cells to cancer tissue^78,79^. Additionally, IL-6 is closely related to cancer-promoting cellular reprogramming and EMT^80^. Although additional research is needed, various proangiogenic factors, including members of the vascular endothelial growth factor A (VEGF-A) and angiopoietin-like protein (ANGPTL) families, are also significantly upregulated in STCs in vitro, which suggests that STCs are closely related to the overall angiogenesis of cancer tissue. Furthermore, a variety of matrix metalloproteinases (MMPs), including MMP1 and MMP9, are upregulated in STCs^17^. SASPs from STCs therefore enormously impact the tumor microenvironment by modulating the immune system, oxygen supply system, and cancer embedding matrix. However, it is well recognized that the role of SASPs is cell type-dependent^16^; the same SASP can have either protumorigenic or antitumorigenic functions depending on the cell type. For instance, IL-6, a well-known protumorigenic SASP in various types of cancer, sometimes acts as an antitumorigenic SASP recruiting antitumor immune cells to the tumor tissue^81,82^. Therefore, it is important to selectively inhibit cancer-promoting SASPs while retaining the antitumor features of SASPs; for example, inactivation of STAT3 reduces protumorigenic SASP without affecting the expression of antitumorigenic chemoattractants in a PTEN-null prostate cancer model^83^.

## Cancer-promoting features of STCs

Considering the potential proinflammatory role of SASPs, senescent cells are widely involved in various aspects of tumor progression^17,18,29^. Since STCs were previously understood to result from defense mechanisms against cancer progression^3,20,48^, other noncancer cell types were the focus of studies initially. Fibroblasts are the most broadly studied cancer-promoting senescent cells in the tumor microenvironment^45–47^. Krtolica et al. first reported that senescent fibroblasts induced the active proliferation of proneoplastic epithelial cells in prostate cancer^45^. Another study also showed that the coinjection of breast cancer cells with senescent fibroblasts led to faster growth of cancer masses in an in vivo mouse xenograft model^84^. Researchers have only recently considered the role of STCs in cancer progression. The first identified cancer-promoting STCs were senescent malignant pleural mesothelioma cells, which were found to increase EMT and chemoresistance in vitro^28^. Pemetrexed-treated senescent mesothelioma cells can induce the expression of EMT-related SASPs, including MMP9 and SNAIL^28^. Moreover, the overexpression of oncogenic H-Ras^G12V^ in normal mesothelial cells resulted in OIS and a similar expression pattern of SASPs and increased EMT^28^.

The first major pathophysiological role of STCs in cancer progression was identified in PTC^17^. IHC analysis revealed that STCs exist in the invasive front of human PTC in a collective manner with a conserved expression of E-cadherin^17^. An in vitro invasion assay also demonstrated that primary STCs had greater invasion ability than non-STCs^17^. Subsequent in vitro leader cell assay, RNA sequencing, and cell migration assay showed that the leading group, SA-β-Gal-positive STCs, releases CXCL12, which in turn attracts other CXCR4-positive non-STCs to invasion sites^17^. STCs were detected in the lymphovascular channels of cancer tissue and survived by increased anoikis resistance in a CXCL12-dependent manner^17^. The presence of STCs in both lymphovascular channels and lymph node metastases in PTC patients suggests that STCs have high metastatic ability^17^.

Another notable feature of STCs is their close relationship with cancer stemness^29^. Cancer stem cells are part of a small population of tumor cells that have self-renewal capacity and differentiate into heterogeneous cell types, and cancer stem cells are significantly involved in cancer recurrence^85^. Karabicici et al. indicated that TIS of cancer cells increases the mRNA expression of stem cell-related molecules, such as CD34 and CD133^86^. Another study showed increased expression of stemness markers in senescent B cell chronic lymphocytic leukemia cells and other human solid tumor cell lines^29^. A p53−estrogen fusion protein experiment found that cancer cells that had experienced cellular senescence had a higher colony formation ability in vitro^29^. The induction of canonical Wnt signaling after chemotherapy is also critical for activating the tumor-initiation ability of STCs^29^.

Recently, we found a very important role of STCs in CRC progression. We observed that STCs also have an enormous impact on the immune system in the tumor microenvironment^18^. Cytotoxic T cells are cancer-killing cells that express death signals, leading to the apoptosis of cancer cells^18^. The importance of cytotoxic T cells in CRC is reflected in the utility of the “Immunoscore”^87^. This score represents the number of infiltrating cytotoxic T cells in tumor tissue and is regarded as a major prognosis-determining factor in CRC patients^87^. An IHC analysis of CRC patients revealed that the number of tumor-infiltrating CD8^+^ cytotoxic T cells was decreased in p16^INK4A^-positive CRC patients^18^. A subsequent in vitro study showed that excess CXCL12, a SASP secreted from STCs, induces the internalization of its receptor CXCR4 in the plasma membrane of cytotoxic T cells^18^. Consequently, cytotoxic T cells lose their localization and cluster around the periphery of the tumor tissue, thus failing to infiltrate the cancer epithelium^18^. The authors also demonstrated that cells positive for CD206, a marker of M2 macrophages, are more likely to infiltrate the cancer epithelium in p16^INK4A^-positive CRC patients^18^. In a subsequent in vitro study, treatment with conditioned media from senescent CRC cells of primary monocytes induced M2 macrophage differentiation, and the effect was confirmed by a coculture system^18^. Furthermore, CSF1 secreted from STCs promoted monocyte differentiation into M2 macrophages, which disrupted cytotoxic T cell activation^18^.

**Fig. 2:**
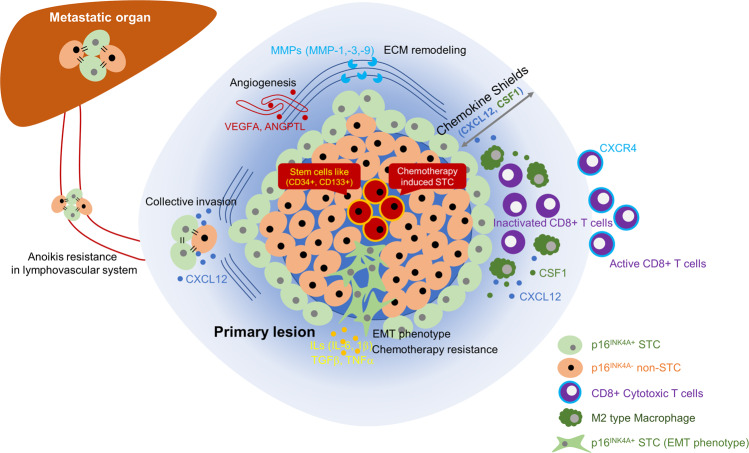
Cancer-promoting features of STCs. STCs participate in cancer progression by releasing various types of SASPs. Three key features are increased cancer invasiveness, enhanced cancer stemness, and a modulated immune cell microenvironment.

In summary, three major cancer-promoting features of STCs are currently known: (1) increased EMT and increased stemness of the STCs themselves, (2) increased release of cancer-promoting factors affecting the invasiveness of nonsenescent cancer cells, and (3) modulation of the local microenvironment, including the immune system, to make it favorable for cancer progression (Fig. 2). Although the amount of evidence for the cancer-promoting qualities of STCs has recently increased^17,18,29,85^, the overall effect of STCs on cancer progression is still not fully understood. STCs are thought to be a group of heterogeneous cells that vary according to the time course of the disease and the underlying induction mechanisms. In other words, their role and effect can be diverse depending on the cancer type and the course of the disease. Therefore, thorough further research considering cancer type and cancer stage is necessary for a full understanding of the physiological role of STCs in cancer.

## Targeting STCs

Based on reports to date, the elimination of senescent cells or STCs from cancer tissues or targeting of SASPs secreted from senescent cells is expected to improve patient prognosis and enhance anticancer treatment^88^. Efforts to selectively eliminate senescent cells for functional rejuvenation have been made by researchers studying aging^89^. The outcome of this vigorous research are agents known as senolytics, which improve aging-related phenotypes and extend patient lifespan by killing senescent cells^88,89^. Various previous trials using senolytics to selectively kill STCs have been conducted. First, dasatinib and quercetin were used to eliminate olaparib-induced senescent ovarian cancer cells^90^. Unfortunately, these senolytcis did not induce the apoptosis of STCs in ovarian cancer cells, and the combination therapy showed low efficacy in removing doxorubicin-induced senescent liver cancer cells^90,91^. Another type of senolytic, ABT263, an inhibitor of the Bcl-2 family, showed a senolytic effect on some types of therapy-induced STCs, including lymphoma cells^92^. However, ABT263 did not show any senolytic activity in prostate or breast STCs^93,94^. Therefore, the effect of senolytics on STCs is cancer-type dependent. Moreover, ABT263 often causes thrombocytopenia, which is the main drawback of clinical application^95^. It is expected that this shortcoming can be overcome by using a modified form of ABT263, a Bcl-xL-directed proteolysis-targeting chimera, which is less toxic to platelets^96^. Similarly, drugs that have senolytic effects on senescent fibroblasts have shown limited effects on STCs or have not been sufficiently studied. For example, piperlongumine, a natural extract that affects radiation-induced senescent fibroblasts, showed a senolytic effect on olaparib-induced senescent ovarian tumor cells^88^. Panobinostat, an FDA-approved histone deacetylase inhibitor, demonstrated senolytic activity in cisplatin- or paclitaxel-induced senescent lung cancer cells^97^. Fisetin failed to show a senolytic effect on olaparib-induced senescent ovarian tumor cells and was also largely ineffective against senescent lung, head and neck, and prostate cancer cells^88,90^. The senolytic effect of the D-retro-inverso isoform of Foxo4 (Foxo4-DRI) and Hsp90 inhibitors, which showed senolytic effects on fibroblasts, on STCs remains to be proven^98^. As advanced drug screening techniques are being developed, conventional drugs used in other fields that are potential senolytics have been discovered. Two independent groups discovered that cardiac glycosides, ouabain, and digoxin are broad-spectrum senolytics by chemical library screening^99,100^. Ouabain had senolytic activity in several drug-induced senescent cancer cells^98^. However, it is noteworthy that supraphysiological concentrations of these agents were used in vitro, which could result in toxicity that is intolerable to patients^101^. The inhibition of glucose metabolism, fatty acid oxidation, and oxidative phosphorylation exerts a senolytic effect in therapy-induced senescent lymphomas^102^. Via unbiased high-throughput screening with oncogene-induced fibroblasts, the senolytic effect of bromodomain and extra terminal domain family protein inhibitors was shown, and the effect that was mediated through the attenuation of nonhomologous end-joining repair and the activation of the autophagic pathway^102^. A recently developed small-molecule BET degrader, ARV825, showed effective senolysis in doxorubicin-induced senescent CRC cells^103^. Drugs disrupting the immune surveillance system also have the potential for eliminating STCs. For example, oxidized forms of membrane-bound vimentin, dipeptidyl peptidase 4, and CD44 were discovered to be senescence-specific surface antigens of senescent mouse lung fibroblasts, human diploid fibroblasts, and endothelial cells, respectively^104–106^. If senescent cancer cell-specific surface antigens are identified, they can be used to deliver cytotoxic drugs or cytotoxic immune cells to senescent cancer cells. In addition, modulating the immunosuppressive SASP of senescent stromal or cancer cells can augment the effects of immune checkpoint inhibitors or other antitumor immunotherapies.

**Fig. 3:**
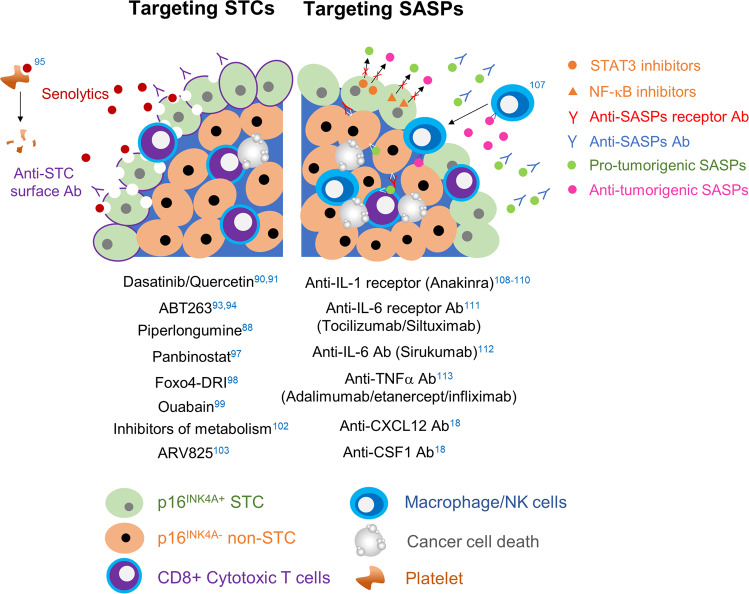
Targeting of STCs *vs*. SASPs. The left and right sides indicate senolytics and senomorphics, respectively. The numbers indicate the corresponding references.

Another type of drug, senomorphics, is being developed to suppress aging by targeting SASPs. Since SASPs are so diverse, targeting their master regulators, such as NF-κB and STAT3, has been considered. However, the transcription factors related to SASP expression are critical for cell survival^76^. Therefore, targeting them can induce critical adverse effects within cells. In addition, since these transcription factors regulate several types of SASPs in a nonspecific manner, some antitumorigenic aspects of SASPs, such as immune clearance of cancer cells via augmentation of immune cell recruitment, can be reduced^107^. Moreover, the major protumorigenic SASPs can be different in various cancers; the same SASP can be either a protumorigenic or antitumorigenic SASP depending on the cancer type^108^. In other words, it might be more effective to inhibit the major tumorigenic SASPs directly^108^. For example, IL-1α/β and IL-1R are known as major signaling axes producing protumorigenic SASPs in oncogene-induced senescent fibroblasts^108^. Without affecting the presence of STCs, IL-1α deletion in K-Ras^G12D^-driven pancreatic cancers in mice has been found to reduce tumor formation and protumorigenic SASP production^109^. Therefore, several therapeutic drugs targeting the IL-1 receptor (anakinra), IL-6 receptor (tocilizumab and siltuximab), IL-6 (sirukumab), and TNF-α (adalimumab, etanercept, and infliximab) have been developed and are currently available in clinical applications for autoinflammatory diseases^110–114^. In line with this, we have shown in a previous study that CXCL12- and CSF1-neutralizing antibodies significantly reduce CRC progression by increasing cytotoxic T cell infiltration in mice with AOM/DSS-induced CRC and increase the effectiveness of immune checkpoint inhibitors, such as PD-1 inhibitors^18^. These drugs could be candidates for selective drug repositioning to inhibit protumorigenic SASPs (Fig. 3).

## Discussion

Although the cancer-promoting features of STCs are continuously being discovered, the overall effect of STCs in cancer tissue on patient prognosis is still disputed. The most challenging limitation to revealing the effect of STCs in the absence of a large cohort study regarding the presence of STCs in cancer. The key reasons making such a study difficult are as follows: (1) the absence of a single specific marker for STC detection and (2) the necessity of several biopsy samples. Since STCs are not evenly distributed in the cancer epithelium, using a single biopsy sample to detect STCs is risky. After a large cohort study with exact patient classification is undertaken, it is expected that a more precise analysis of STCs can be performed. Another barrier for the STC study is the absence of an appropriate in vivo model. Tumor mouse models for identifying STCs in human tumors have not been sufficiently validated. Therefore, most of the research comparing the physiological roles of STCs and non-STCs has been conducted in vitro and is thus limited. Some researchers have used an in vivo on−off switch system to compare STCs and non-STCs, but the methodological variance is still lacking. It will be interesting to construct cell-type-specific p16^INK4A^-ATTAC and p16^INK4A^-3MR mice to determine the role of STCs in various mouse cancer models and to discover important protumorigenic SASPs in each cancer type^115^.

Although additional studies should be performed with various cancer tissues, previous studies have shown that STCs are more closely related to cancer progression than to cancer inhibition^17,18,28,29,84^. Moreover, these cancer-promoting features are caused by SASP secretion from STCs^17,18^. Considering the adverse effect of current senolytics on normal cells and patients, a new generation of senolytics should be developed for cancer therapy^95^. Presently, targeting STC-derived SASPs, including IL-1, IL-6, TNF-α, CSF1, and CXCL12, is the most appropriate therapeutic strategy to control STCs.
